# Supplementary material for: Carbon losses from deforestation and widespread degradation offset by extensive growth in African woodlands
Source: Nat Commun. 2018 Aug 2;9:3045. doi: 10.1038/s41467-018-05386-z (PMC6072798; doi:10.1038/s41467-018-05386-z)
Supplement: Supplementary file 1 — Supplementary Information [file 41467_2018_5386_MOESM1_ESM.pdf]

Supplementary information

Carbon losses from deforestation and widespread degradation offset by extensive growth in African woodlands

McNicol et al.

Supplementary Figures 2
Supplementary Tables 12
Supplementary methods 16
  In situ carbon stock estimates 16
  Estimating aboveground woody carbon stocks 17
  Estimating biomass carbon stocks, carbon stocks changes and rates of land cover change . 18
  Estimating uncertainty 19
  Soil moisture correction 20
  Masking of non-wooded and flooded land 21
Supplementary References 23

Supplementary Figures

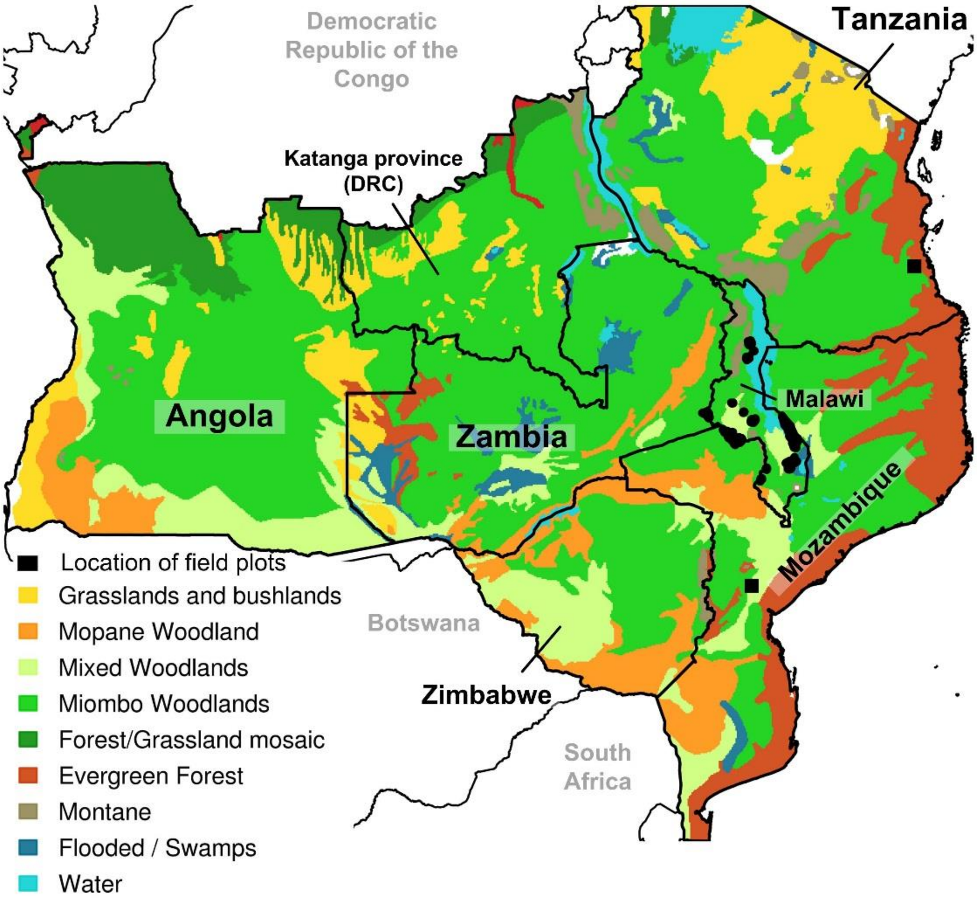

Supplementary Figure 1 | Study region vegetation types. The spatial distribution of major vegetation types across our study region, adapted from White's vegetation map of Africa[1]. The original data set is based on a combination of pre-satellite era vegetation maps and expert opinion, and contains more detailed information of the floristic and structural characteristics of the vegetation within a given area. This includes distinguishing between different types of 'miombo' woodlands, including those dominated by *Brachystegia* and/or *Julbernardia* , and/or those occurring in wetter (>1000 mm) parts of the study region. For simplicity, here, vegetation types were combined according their physiognomy and key structural characteristics (e.g. closed vs. open canopy, tree dominated vs. grass dominated).
The location of the field plots used to calibrate the radar data are shown as black points.

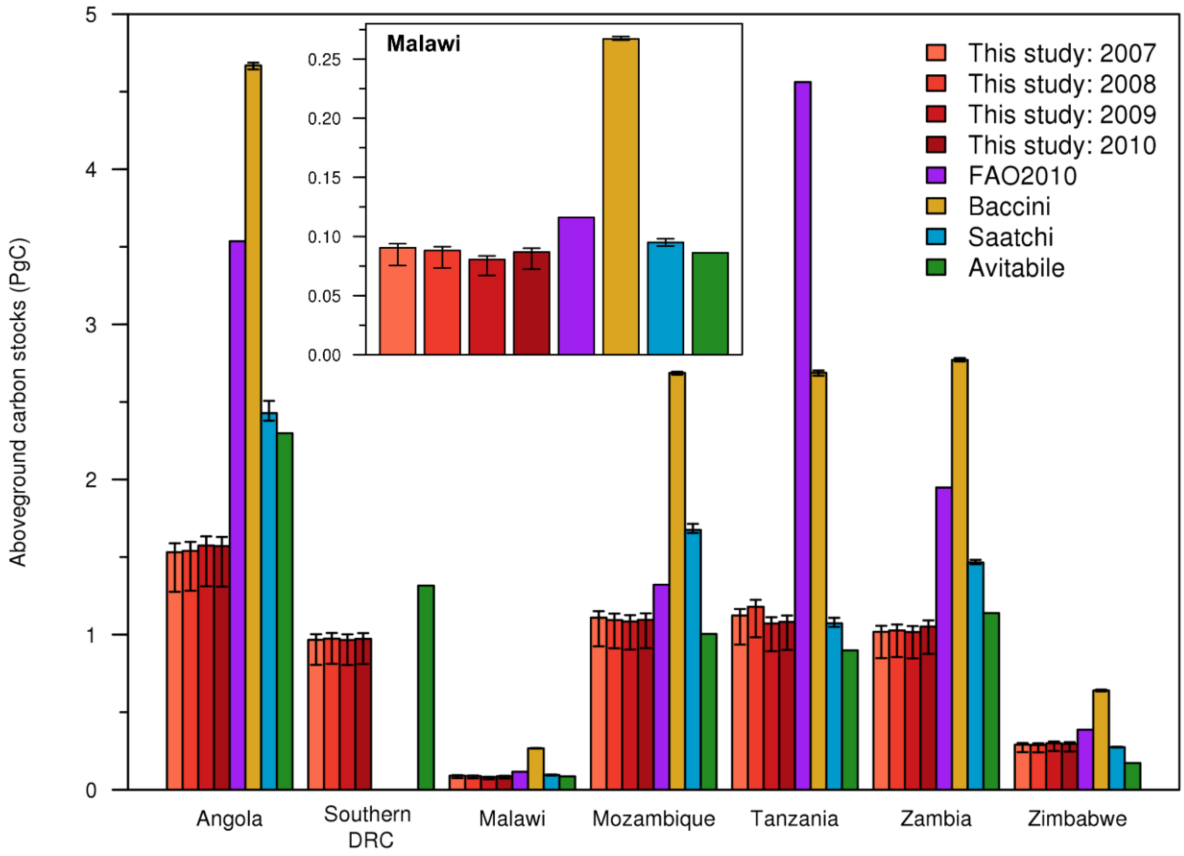

Supplementary Figure 2 | Comparisons of AGC stocks between years, and to existing estimates. The national-level carbon stocks in each year of our study, alongside the data obtained from several other studies, including any reported uncertainties (Saatchi). The inset figure is more detailed comparison of the AGC stock data for Malawi. The error bars on our estimates represent the 95% confidence bounds, and are described in the Supplementary Methods section on ‘Estimating Uncertainty’.

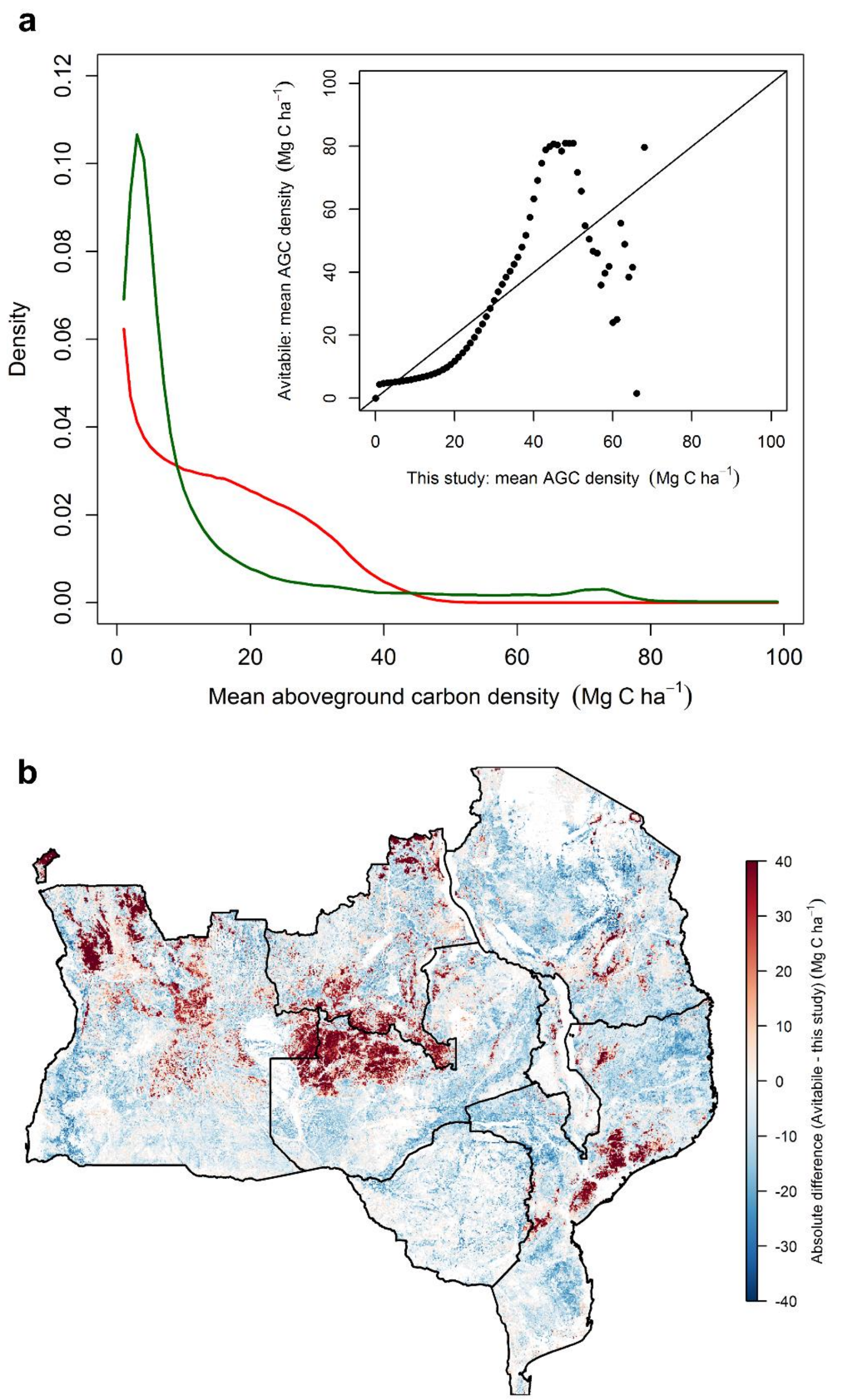

Supplementary Figure 3 | Detailed comparison of the Avitabile et al. (2016) AGC map and our 2007 map, aggregated to 1 km resolution. (a) Histograms of the biomass distributions for the two maps (red = this study, green = Avitabile) with the inset figure showing the average AGC density of cells in the Avitabile dataset compared to the corresponding AGC densities in our study, in 1 MgC ha^{-1} bins. (b) The absolute difference (Avitabile – our study AGC_{2007}) in average AGC densities in each 1km cell.

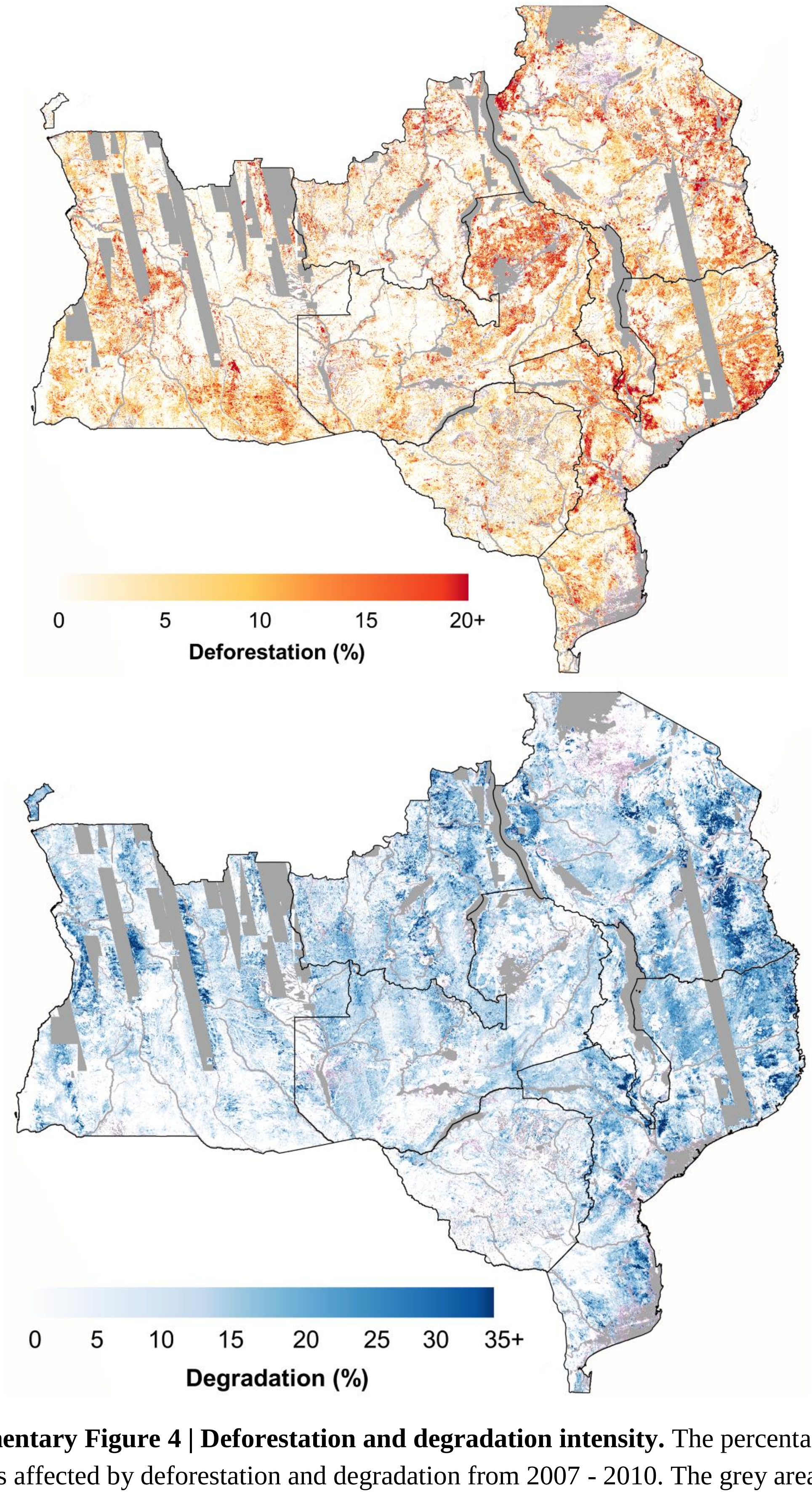

Supplementary Figure 4 | Deforestation and degradation intensity. The percentage of 1 km pixels affected by deforestation and degradation from 2007 - 2010. The grey areas indicate areas masked form the analysis.

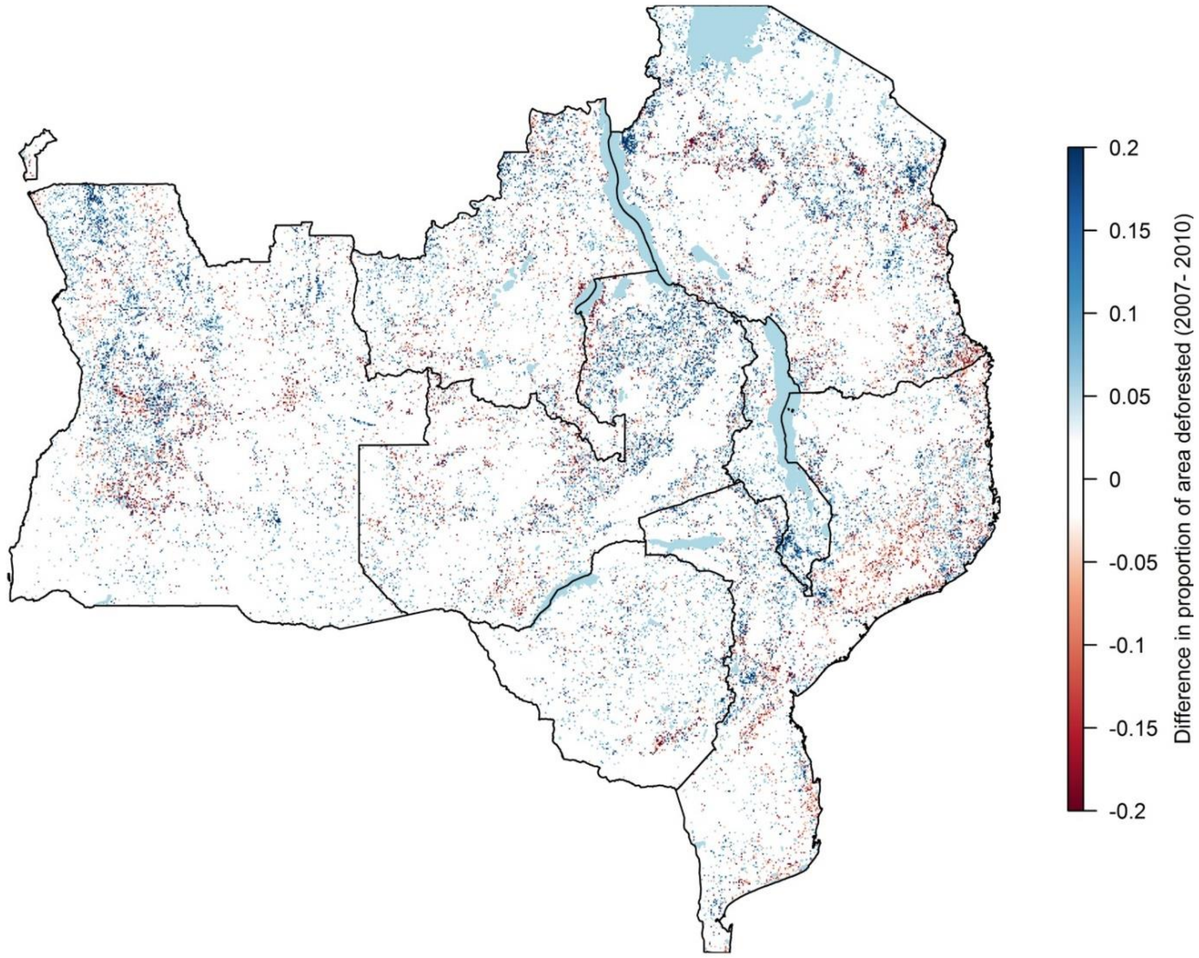

Supplementary Figure 5 | Spatial comparison of our dataset and the Hansen dataset. The difference the Hansen et al. (2013) deforestation map and our dataset with positive values (blues) indicating that our study detected higher area of deforestation within a 9 ha pixel, and negative values (red) indicating greater deforestation in the Hansen dataset. Differences were found to be most pronounced in areas with relatively low woody cover and biomass in 2007, with our study detecting considerably greater deforestation in these more open areas, including large parts of Malawi and the north-eastern parts of Zambia and Tanzania. Hansen detects greater deforestation in Zambezia province in central Mozambique, and parts of Angola.

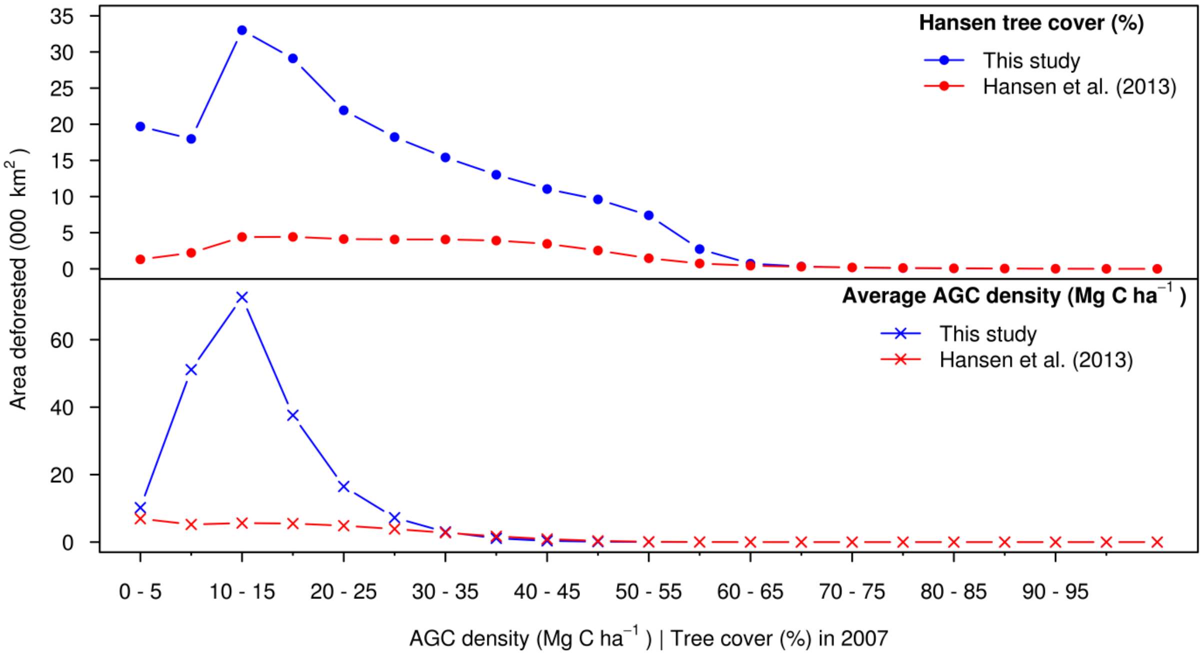

Supplementary Figure 6 | Comparisons of the location of deforestation in this study and the Hansen dataset according to the initial AGC density and tree cover. The area of deforestation detected by this study (blue symbols and lines) and by Hansen (red symbols and lines) according to the average AGC density in 2007 as measured in this study (solid lines), and the average tree cover (%) in 2007 as measured by Hansen (hatched lines). The data is separated into bins of 5 MgC ha^{-1}, or percentage tree cover.

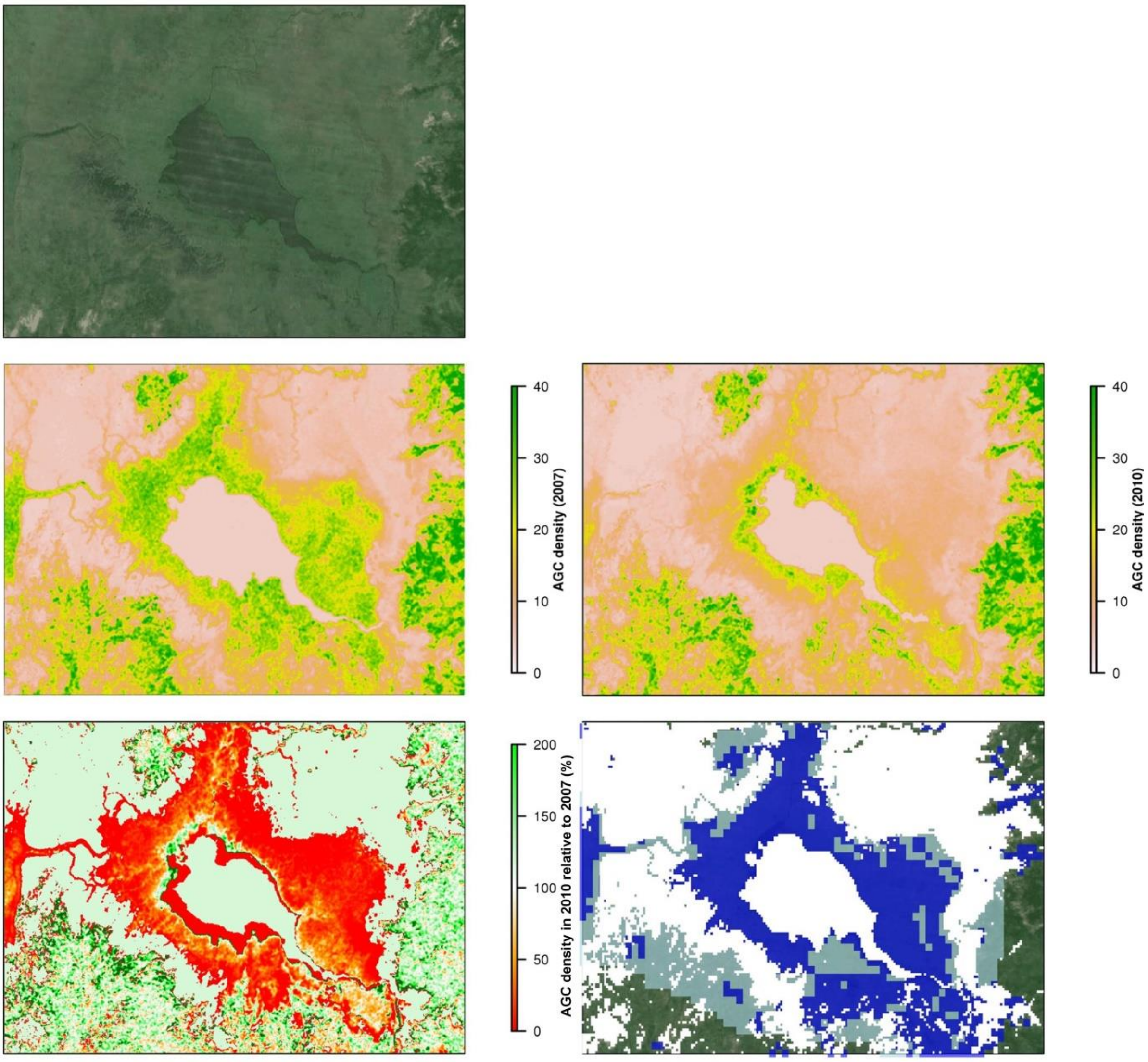

Supplementary Figure 7a | Example of land use and flooded area mask. False change detections around periphery of lakes were masked from the analysis. An example of this is shown for Lake Urema (-18.33, 35.99) in central Mozambique with the middle two panels in the above figure showing the associated biomass estimates for 2007 (left) and 2010 (right). The widespread reductions in biomass displayed in the initial change map (2010 / 2007), shown in the bottom-left panel, are due to soil moisture changes in the grassland floodplain. The darker blue shades in the bottom-right panel indicate flooded or swampland areas according to the ESA land cover maps meaning these areas were masked out, while the lighter blue indicate the additional buffer zone around these areas to account for likely false changes not accounted for in when using the wetland mask.

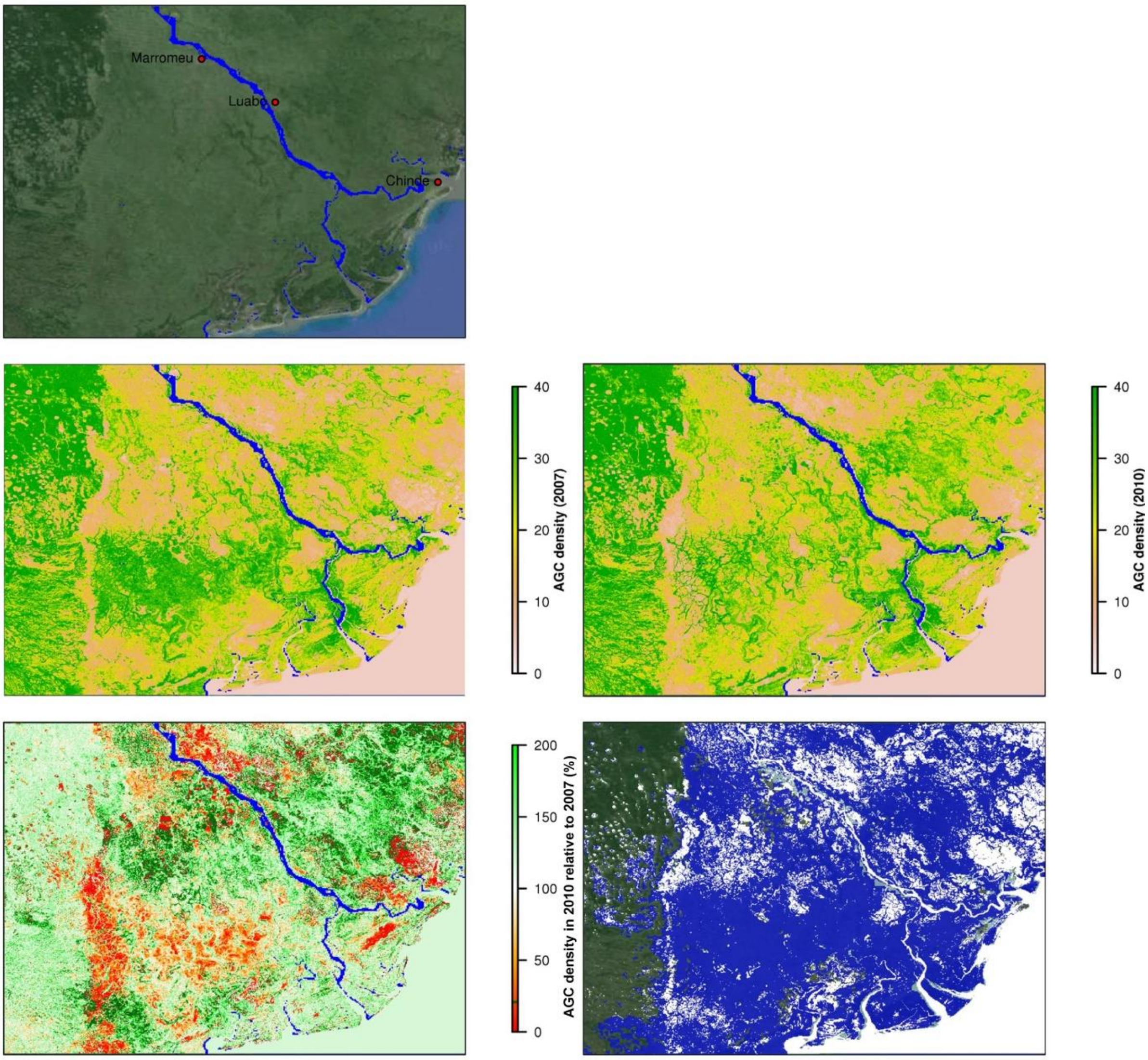

Supplementary Figure 7b | Example of land use and flooded area mask. The areas around the edges and floodplains of large rivers, such as the Zambezi delta as shown in top image (river in blue according the ESA's LCC maps), were also masked out in order to avoid potential seasonal waterlogging resulting in false change detections. Again, clear differences in biomass can be observed between 2007 (middle-left) and 2010 (middle-right) with large areas in the associated change map (bottom-left; (AGC_{2010} / AGC_{2007}) ×100) being classified as either highly degraded or deforested, while other areas appeared to have considerably gained biomass (green) with some areas appearing to have at least doubled in biomass over the 3 year period (darker greens). However these changes are artefacts caused by soil moisture changes in the delta. The darker blue in the bottom-right panel again indicate areas identified as either flooded or swampland in the ESA land cover maps. Areas with an AGC density < 10 MgC ha^{-1} in 2007 are shown in white.

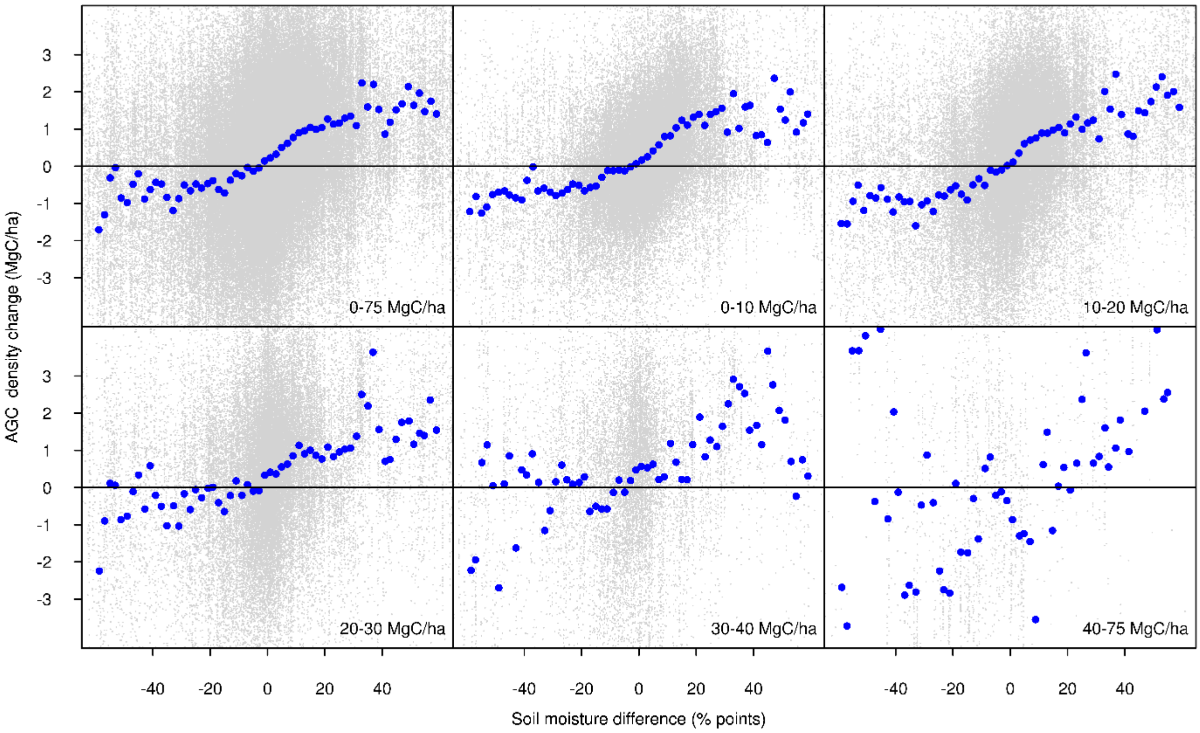

Supplementary Figure 8 | AGC change by the soil moisture difference between 2007 and 200. The relationship(s) between the change in soil moisture between 2007 and 2010, and the estimated change in biomass - converted from backscatter for clarity - using the full dataset, and then after subdividing the dataset into five different levels of AGC stock. Both datasets were aggregated to 5 km resolution to reduce the likelihood of land cover changes affecting the results. The change in soil moisture is calculated by subtracting the 2007 values from the 2010 data. As such, negative values indicate a wetter 2007, which result in false observation of biomass decreases, whereas positive changes in soil moisture indicate a wetter 2010, favour biomass increases. Blue dots show the mean for each 1 MgC ha^{-1} bin, whilst grey shows the observations.

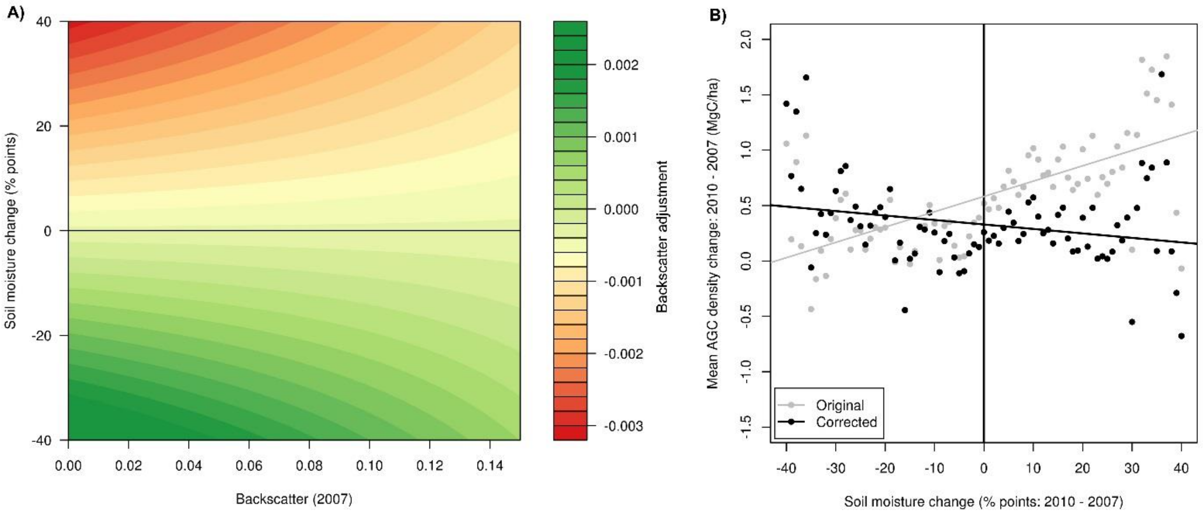

Supplementary Figure 9 | Illustrative example of soil moisture correction and the effect of the correction on the backscatter data. (A) The adjustment to the observed backscatter change, for soil moisture changes at different levels of backscatter in 2007. The difference in soil moisture is calculated by subtracting the 2007 values from the 2010 data. (B) Estimated biomass change as a function of soil moisture, before (grey; $y = 0.0134x + 0.58$, $r^2 = 0.38$, $p < 0.05$) and after (black; $y = -0.004x + 0.32$, $r^2 = 0.04$ $p = 0.04$) the new correction has been applied. The positive trend which characterised the raw dataset has been removed. For clarity, the y-axis is rescaled to AGC density changes (MgC ha^{-1}).

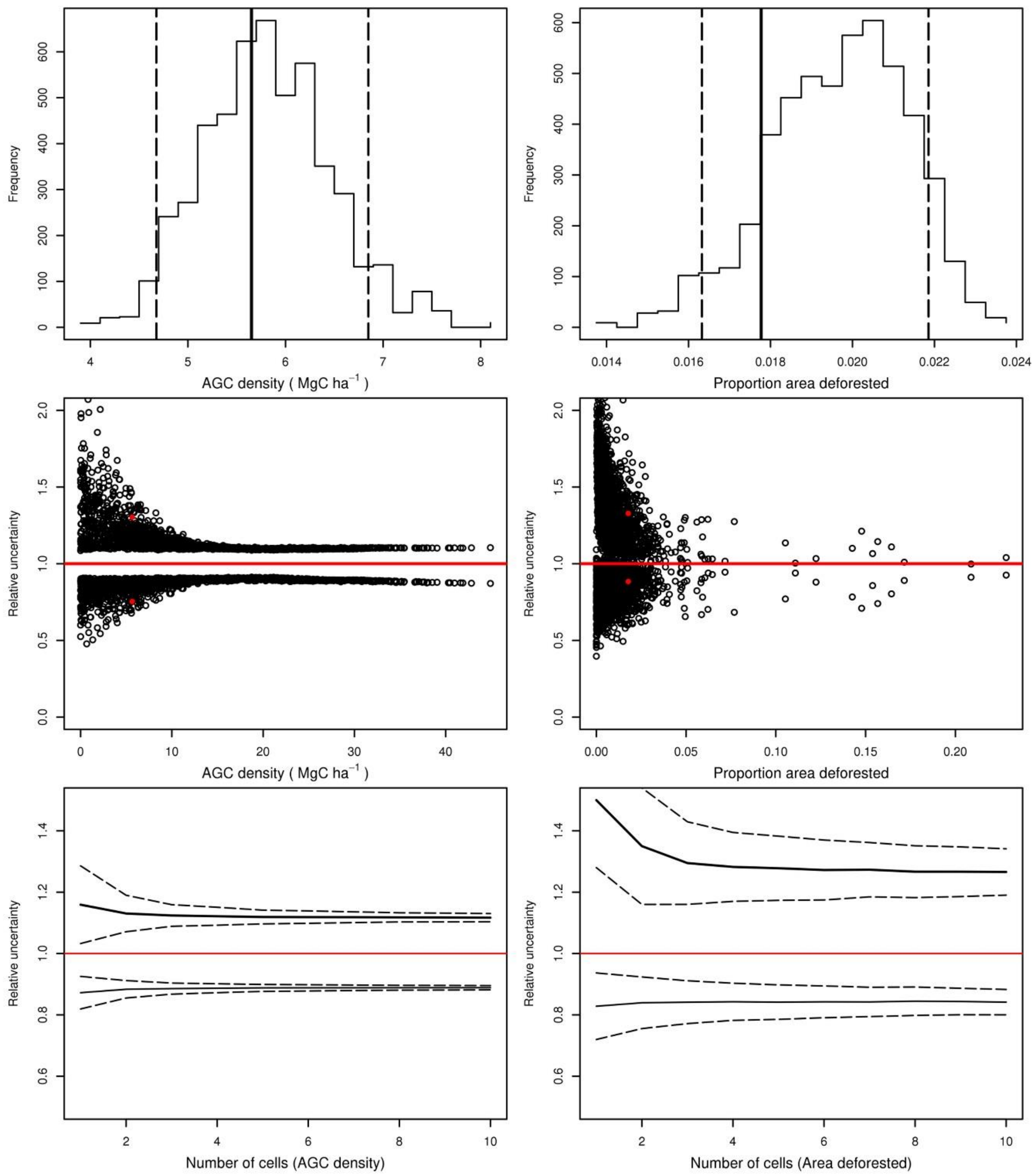

Supplementary Figure 10 | Estimating uncertainty on all derived quantities. The top panels show the variation in estimated AGC stocks in 2007 (top left) and deforestation rates (top right) for one of the 2000 cells after repeating the analysis using 5000 biomass-backscatter models. The solid vertical lines correspond to the estimate derived using the best-guess biomass-backscatter model (Eq .1), with the hatched lines indicating the 2.5 and 97.5 percentiles of the 5000 estimates, which represent the uncertainty bounds on our estimates for that area. The middle panels show these error bounds (%) as a function of the original prediction across the 2000 squares with the red points indicating the data from the top panels. The bottom panels show the effect of combining a progressively larger number of the 100 km^2 squares on the relative error on AGC stocks (bottom left) and the area deforested (bottom right) illustrating the scale dependence of the error.

Supplementary Tables

Supplementary Table 1 – Model coefficients and goodness of fit statistics for the biomass-backscatter relationship for each area and the general model using all data. Numbers in brackets indicate standard error.

Dataset	Slope	Intercept	R^2	Bias (MgC ha^{-1})	RMSE (MgC ha^{-1})
**Tanzania**	633 (78)	1.31 (2.8)	0.74	2.28	6.86
**Mozambique**	757 (73)	-8.2 (2.4)	0.53	1.37	8.35
**Malawi**	946 (244)	-17.8 (11.4)	0.47	4.32	11.75
**General**	716 (54)	-5.97 (1.90)	0.57	1.17	8.51

Supplementary Table 2 - Confusion matrix of the Hansen deforestation dataset and our radar-derived maps of deforestation and degradation. Values represent the number of 9 ha cells that meet the criteria. The column labelled as 'Masked' refers to cells that were completely masked (100%) in this study, whereas the column entitled 'Partial Masking' refers areas where only some of the 9 ha cell was masked (<100%).

		**This study**				
		Deforestation	Degradation	Masked	Partial masking or minor carbon losses or gains	Total
**Hansen**	Deforestation	4,428,832	1,607,058	568,171	939,278	7,543,339
	No Deforestation	18,674,883	11,440,966	4,695,499	10,470,045	45,281,393
	Total	**23,103,715**	**13,048,024**	**5,263,670**	**11,409,323**	52,824,732

Supplementary Table 3 – A comparison of the most recent estimates of AGC stock changes in southern Africa

Dataset	Gross losses (PgC)	Net AGC change (PgC)	Scope
This study	0.29 (0.34)*	+0.024	Angola, Malawi, Mozambique, Tanzania, Zambia, Zimbabwe & (former) Katanga province (DRC)
FAO FRA	--	-0.38**	As above, but excluding Katanga
Hansen + Avitabile	0.048	--	Same as this study
Grace et al. (2016) (Hansen + Saatchi)	0.36 - 0.54**	--	Tropical Africa

* Losses due to Deforestation to Degradation only, with those in brackets including minor losses

** Between 2005 and 2010

*** Assumes degradation losses are 10 - 50% of deforestation

.

Supplementary methods

In situ carbon stock estimates

Radar backscatter is not a ‘direct’ measure of woody biomass [2], and is instead related to the density and size of the vegetation (which tend to be correlated with biomass) and its dielectric properties, as well as the roughness and moisture content of the soil. Therefore, in order to estimate aboveground carbon densities from radar data, backscatter values were regressed against field measured carbon stock data taken from 120 woodland inventory plots in Tanzania and Mozambique and 17 forest reserves in Malawi.

The Tanzanian plots are located in Kilwa District, Lindi Region, and consist of 24 × 1 ha (100 × 100 m) plots set up in 2010 - 2011 as part of a REDD+ pilot project [3]. The Mozambican plots were established between 2006 and 2010 and are described in Ryan et al., (2011, 2012), and include 96 plots ranging from 0.1 – 2.2 ha in size. For Malawi, 277 × 0.1 ha plots were available and located in 17 forest reserves which varied in size from 6 km^2 to 989 km^2 (Makungwa, 2012). At the Tanzanian and Mozambican sites all trees with a diameter at breast height (DBH) >5 cm were measured, whereas in Malawi, a nested sampling strategy was employed, with stems 5 – 20 cm measured within a central 0.01 ha circle plot, and stems >20 cm DBH recorded in the surrounding 0.1 ha plot, adjusted to account for the slope of the terrain. Smaller plots are difficult to relate to radar data due to speckle, geolocation errors and the variability in woody structure [5]. Thus for the Malawi dataset, the mean AGC density of each forest reserve was calculated as the mean of all the 0.1 ha plots located each reserve (mean n = 22, range = 6 - 57). For the remaining sites, the median and mean plot size is ~0.6 ha, with a mode of 1 ha.

For consistency, the biomass carbon stock of trees at all sites was estimated using a DBH-only allometric model developed near to the Mozambican plots, which comprise the majority of the field data used for calibration [4]. The choice of allometric equation typically adds a systematic bias to the estimates of woody biomass on a set of plots, which will be propagated to the AGC maps. We quantified this bias by re-calculating plot AGC stocks using three alternative allometric models; the pan-tropical models of Chave et al. (2005, 2014), and a model developed in Tanzanian woodlands [6]. In each instance, we use the best predictive model that does not include tree height as it was not systematically collected across all our plots. For the Chave models, we use a single wood density value that is based on the average density of the dominant trees at the Mozambican site [7].

Estimating aboveground woody carbon stocks

To convert backscatter images in to maps of AGC, the mean backscatter of each plot in the year closest to the time of the field inventory was regressed against its field-estimated carbon stock using ordinary least squares (OLS) regression. Both linear and 2nd order polynomial models were fitted, the latter a check for saturation in the relationship. Analysis of Covariance was used to evaluate whether models from each country were significantly different. We employed a 5000 x 2-fold cross validation procedure, withholding half of the biomass-backscatter dataset to estimate the validation Root Mean Square Error (vRMSE) and validation bias on regression models fit using the remaining half of the data.

There was a moderate to strong relationship between AGC stocks and radar backscatter for each of the three datasets: OLS R^2 = 0.47, 0.53 and 0.74 for Malawi, Mozambique and Tanzania, respectively (Supplementary Table 1). Linear models were favoured over 2nd order polynomials (quadratic term $P > 0.2$ in all cases) indicating no saturation in the relationship. An Analysis of Covariance indicated no significant difference in the slope coefficient for the three site-specific models ($P = 0.32$), but a significant difference in the intercept of the model fit to the Tanzanian dataset, compared those fit to the data from Mozambique and Malawi (P = <0.05). However, a general model using data from all sites resulted in lower validation bias, which is the key criteria for wide area mapping of biomass [8]. Thus, a general model using data from all plots and plot groups (n = 137) was used to estimate biomass from backscatter:

$$\text{AGC} = 715.7 \times \gamma^0 - 5.97 \qquad (1)$$

$$R^2 = 0.57;\ P < 0.001$$

A linear model again outperformed the quadratic fit (quadratic term p = 0.2). The cross validation procedure indicated a mean vRMSE of 8.5 ± 1.5 MgC ha^{-1} and a bias of 1.1 ± 0.3 MgC ha^{-1} for the general model (see Supplementary Table 1). The vRMSE, which represents the error on a prediction of AGC for a single pixel, suggests that small changes in AGC will be difficult to detect for an individual pixel, however, these errors cancel out when estimating changes in biomass over many pixels. Indeed, if we assume the errors in each year are uncorrelated (the most conservative scenario), then the RMSE for an area covering 100 pixels (n) is given as sqrt(RMSE^2 × n). If the area had the mean AGC density of 23.8 MgC ha^{-1} then this error is 3.6% of the total carbon stock; if 10,000 pixels are considered, the error reduces to 0.36% of the stock. Thus, the RMSE starts to become trivial for progressively larger areas, even though it is important for a single pixel.

Regional estimates of AGC storage were 18% higher when using the Mugasha et al. (2013) model, and 11% higher when using the Chave et al. (2005) model to predict plot AGC stocks. Hence, our use of the Ryan et al. (2011) model results in lower estimates of AGC storage (and thus losses) than the best alternative regionally developed model. A similar estimate (-5% lower) was produced when using the pan-tropical model of Chave et al. (2014), which includes the raw data from both the Ryan et al. (2011) and Mugasha et al. (2013).

Estimating biomass carbon stocks, carbon stocks changes and rates of land cover change

AGC stocks, AGC changes and the areal extent of the LCCs are presented at the national level, and the two lower administrative units, termed regional ('province' in Angola, Mozambique and Zimbabwe; 'district' in DRC) and district level ('municipality' in Angola; 'commune' or 'territory' in DRC) (Supplementary Data 1). Carbon stocks were calculated by converting pixels from carbon densities (MgC ha^{-1}) into carbon stocks (MgC) and then summing within each administrative unit. Carbon stock changes between 2007 and 2010 include the total carbon gains (in non-masked areas wooded in 2007), total carbon losses and the carbon stock change attributable to each LCC.

The carbon stock changes (ΔB) associated with each land cover change were estimated as the per pixel product of the observed stock change and the probability that each LCC had occurred. Thus:

$$\Delta B_{\text{deforestation}} = [B_{10}\text{-}B_{07}] \times P(\text{deforestation}) \quad (2)$$

$$\Delta B_{\text{degradation}} = [B_{10}\text{-}B_{07}] \times P(\text{degradation}) \quad (3)$$

$$\Delta B_{\text{gain}} = [B_{10}\text{-}B_{07}] \times P(\text{gain}) \quad (4)$$

$$\Delta B_{\text{minor loss}} = [B_{10}\text{-}B_{07}] \times P(\text{minor loss}) \quad (5)$$

Where ΔB is the change in biomass in MgC ha^{-1} over the three years, B is the biomass stock, and $P()$ is the probability of the land cover change occurring. In the above example where a pixel moved from 25 to 16 MgC ha^{-1}, we would calculate a weighted carbon loss due to degradation of 6.75 MgC ha^{-1} (9 MgC ha^{-1} × P(degradation) = 0.75). The area affected by each LCC, and the total wooded and non-wooded area, was obtained by summing the relevant probabilities, or weighted estimates over the area of interest as follows:

$$\sum_{i=1}^{n} P(\text{wooded})_{i \ldots n} \quad (6)$$

$$\sum_{i=1}^{n} P(\text{deforestation})_{i \ldots n} \quad (7)$$

$$\sum_{i=1}^{n} P(\text{degradation})_{i \dots n} \quad (8)$$

$$\sum_{i=1}^{n} P(\text{gain})_{i \dots n} \quad (9)$$

$$\sum_{i=1}^{n} P(\text{minor loss})_{i \dots n} \quad (10)$$

Where n is the number of pixels within the area of interest. The rates of LCC changes were calculated by dividing the affected area by the area of woody cover in 2007, and then dividing by 3 to account for the number of years the data covers to produce a mean annual rate of change.

Estimating uncertainty

The uncertainties on our carbon stock and change estimates, and the land cover change probabilities were calculated by repeating the analysis using a different one of the 5000 biomass-backscatter models generated during the cross-validation procedure (described in section 2.3). For each model, we create new AGC maps and LCC probability matrices (Supplementary Figure 3), and re-calculate all the derived quantities reported here. Due to computational limitations, this process was performed on 5% of the study area, comprised of 2000 randomly selected 10 km x 10 km squares. For each area, we calculate all derived quantities, retaining the 2.5th and 97.5th percentiles of the 5000 estimates, and use these 95% CI to approximate the uncertainties over the whole study area.

The relative uncertainty on the estimated biomass stocks and change statistics – calculated as a proportion of the original, "best-guess" predictions in each square – was not consistent across the 2000 squares, typically being higher and more variable in areas of low biomass or with little LCC, largely due to the small absolute values leading to large relative errors. This effect is scale dependant, as over larger areas the absolute values are larger (e.g. it became less and less likely that the area deforested will be very small); hence we evaluated the scale dependence by combining a progressively larger random subsample of the 2000 squares, with each increase repeated 5000 times so that a different combination of grid cells were used. The results revealed a reduction and then saturation in the relative error of all quantities as the area considered increases (Supplementary Figure 10). For aboveground carbon stocks, the 95% CI ranges from 89 – 111% of the best-guess stock estimate at scales of >400 km^2, whereas for the deforestation rate, the 95% CI ranges from 84 – 124%, saturating above 600 km^2. We use these saturation values as the uncertainty on all quantities reported here, as they are applicable at the scales considered here, with the exception of some very small districts where the uncertainty may be higher (Supplementary Data 1). In

summary, our uncertainty estimates are valid for areas > 600 km^2, but in smaller areas, particularly those with low carbon stocks or low rates of change, the uncertainty will be higher than estimated.

Soil moisture correction

L-band radar backscatter is somewhat sensitive to several environmental parameters unrelated to woody biomass, particularly soil moisture [10]. To minimise this problem when estimating biomass, dry season imagery is commonly used [8,11,12]; however the ALOS PALSAR mosaic product includes some images acquired outside the dry season (data is from April – December; dry season typically from May- Nov), meaning that some wet season imagery is present in the data. This seasonal soil moisture effect, combined with the effect of the local hydrology of drainage lines and flood plains (see next section) needs to be accounted for in multi-temporal woody biomass estimation.

To account for seasonal moisture effects, we developed a statistical model that applies a small correction to the backscatter data in areas where the estimated soil moisture varied between years. The model provides for differential corrections according to the estimated woody biomass, following the logic of the Water Cloud Model [13]. It was constructed by parameterising the following regression model in R statistical software:

$$\Delta\gamma^0 \sim \Delta\vartheta + \Delta\vartheta \times B_{07} \qquad (11)$$

Where $\Delta\gamma^0$ is the change in radar backscatter between 2007 and 2010, $\Delta\vartheta$ is the associated change in soil moisture (%), and B_{07} is the backscatter in 2007.

Soil moisture data were obtained from the European Space Agency's (ESA) ECV soil moisture product (http://www.esa-soilmoisture-cci.org; Liu et al. 2011). The soil moisture data is provided at a global scale at a spatial resolution of 0.25 degrees, with satellite return interval of 2-3 days. We used the active soil moisture product which is based on data obtained from AMI-WS and ASCAT C-band scatterometers on board the ERS-1, ERS-2 and METOP-A satellites and provides daily estimates of percentage saturation (%). For each pixel covering the study region we extracted the soil moisture values for all dates where data was available between 2007 & 2010, along with the associated acquisition date(s) of all the radar data collected for that area. We interpolated along the soil moisture time-series using a 7-day moving average to allow the prediction of soil moisture in areas where no moisture data was available on the associated radar acquisition date.

The effect of changes in soil moisture on estimated biomass changes is visualised in Supplementary Figure 8. The comparison revealed that reductions in backscatter tended to be more frequently observed in areas where soil moisture decreased between 2007 and 2010, with increases more prevalent in areas where the soil moisture was greater in 2010, although the magnitude of the effect tends to be small (~ 1 MgC ha^{-1}), with the trend diminishing in areas with higher AGC densities in 2007, as predicted by the WCM. Extreme changes in moisture (e.g.> 40% points) resulted in some large, idiosyncratic changes in backscatter, most notably in Central Angola where there was some notable striping present in the data, although such areas comprise only 5% of the dataset. These areas were therefore excluded from both the analysis (5% of the study region), and the statistical model, which was fit using only the data where moisture varied by less than this value. The parameters of the resultant model was used to apply a small correction to the backscatter maps from 2008 – 2010, with the size of the adjustment decreasing as AGC stocks increase (Supplementary Figure 9):

$$\Delta\gamma^0 = 0.0003528 + 0.00006013\ \Delta\vartheta - 0.00006387\ \Delta\vartheta \times B_{07} \qquad (12)$$

Where $\Delta\gamma^0$ is the backscatter adjustment, $\Delta\vartheta$ is again the associated soil moisture change, and B_{07} is the backscatter in 2007. The correction successfully resulted in the minor effect of soil moisture being almost fully removed from the AGC change estimates (Supplementary Figure 9).

Masking of non-wooded and flooded land

The relatively coarse resolution of the soil moisture dataset (25 km x 25 km resolution) means that finer scale soil moisture effects, for example around drainage lines and flood plains, are unlikely to be fully accounted for in the soil moisture correction detailed above. To this end we developed a mask layer to exclude areas where the biomass-backscatter relationship is likely to be invalid (Supplementary Figure 7). Urban areas (where the radar signal interacts with buildings) and other non-vegetated land covers can also create artefacts in biomass estimation and need to be masked from the analysis.

Water bodies (buffered by 1.2 km to account for seasonal flooding), irrigated croplands, flooded and urban areas were masked based on the European Space Agency's (ESA) Climate Change Initiative (CCI) Land Cover product [15]. We also masked rivers and drainage lines (again, buffered by 1.2 km), with a 3 km buffer applied to large rivers (from Digital Chart of the World), and 600 m buffer for smaller drainage lines not captured in the ESA land cover product (from Hansen et al. 2013). We also exclude non-wooded lands (<10 MgC ha^{-1} in

2007) from our change analysis as soil effects in these areas are likely to be strong (Lucas et al. 2010; Ryan et al. 2012; Tanase et al. 2014). At the opposite end of the AGC gradient, any pixel with an AGC density >75 MgC ha^{-1} was capped at this value in order to account for the decrease in sensitivity of L-band radar at higher AGC values leading to an eventual saturation in the backscatter response [17]. Varying the cap from 60 MgC ha^{-1} (the highest field measured AGC value) to 100 MgC ha^{-1} had a negligible effect on AGC stocks and rates of LCC owing to the small number of these high biomass pixels. Areas with a backscatter of >0.15 m^2/m^2 (-8.2 dB) in any year, equivalent to an AGC density of >100 MgC ha^{-1}, were completely removed from the analysis as AGC values of this magnitude are considered ecologically unrealistic for the majority of the region, and based on our observations, tend to be the result of undocumented urban areas and on steep slopes and hill crests, meaning that masking was favoured over capping these areas at 75 MgC ha^{-1} [4,18,19].

Overall, our data mask of urban areas, irrigated cropland, lakes, rivers, swamps and the associated buffers covers 11% of the total land area, with areas with a backscatter >0.15 m^2/m^2 (-8.2 dB) comprising an additional 2%, with the soil moisture change mask excluding an additional 5%.

Supplementary References

1. White, F. The Vegetation of Africa: A Descriptive Memoir to accompany the Unesco/AETFAT/UNSO Vegetation map of Africa. *UNESCO Nat. Resour. Res. Rep.* **20,** 1–356 (1983).
2. Woodhouse, I. H., Mitchard, E. T. a, Brolly, M., Maniatis, D. & Ryan, C. M. Radar backscatter is not a 'direct measure' of forest biomass. *Nat. Clim. Chang.* **2,** 556–557 (2012).
3. McNicol, I. M., Ryan, C. M., Dexter, K. G., Ball, S. M. J. & Williams, M. Aboveground carbon storage and its links to forest structure, tree species diversity and floristic composition in south-eastern Tanzania. *Ecosystems* (2017). doi:10.1007/s10021-017-0180-6
4. Ryan, C. M., Williams, M. & Grace, J. Above- and Belowground Carbon Stocks in a Miombo Woodland Landscape of Mozambique. *Biotropica* **43,** 423–432 (2011).
5. Réjou-Méchain, M. *et al.* Local spatial structure of forest biomass and its consequences for remote sensing of carbon stocks. *Biogeosciences Discuss.* **11,** 5711–5742 (2014).
6. Mugasha, W. A. *et al.* Allometric models for prediction of above- and belowground biomass of trees in the miombo woodlands of Tanzania. *For. Ecol. Manage.* **310,** 87–101 (2013).
7. Williams, M. *et al.* Carbon sequestration and biodiversity of re-growing miombo woodlands in Mozambique. *For. Ecol. Manage.* **254,** 145–155 (2008).
8. Ryan, C. M. *et al.* Quantifying small-scale deforestation and forest degradation in African woodlands using radar imagery. *Glob. Chang. Biol.* **18,** 243–257 (2012).
9. Chave, J. *et al.* Tree allometry and improved estimation of carbon stocks and balance in tropical forests. *Oecologia* **145,** 87–99 (2005).
10. Motohka, T., Shimada, M., Uryu, Y. & Setiabudi, B. Remote Sensing of Environment Using time series PALSAR gamma nought mosaics for automatic detection of tropical deforestation : A test study in Riau , Indonesia. *Remote Sens. Environ.* **155,** 79–88 (2014).
11. Lucas, R. *et al.* An evaluation of the ALOS PALSAR L-band backscatter - Above ground biomass relationship Queensland, Australia: Impacts of surface moisture condition and vegetation structure. *IEEE J. Sel. Top. Appl. Earth Obs. Remote Sens.* **3,** 576–593 (2010).
12. Joshi, N. *et al.* Mapping dynamics of deforestation and forest degradation in tropical forests using radar satellite data. *Environ. Res. Lett.* **10,** 34014 (2015).
13. Attema, E. P. W. & Ulaby, F. T. Vegetation modeled as a water cloud. *Radio Sci.* **13,** 357 (1978).
14. Liu, Y. Y. *et al.* Developing an improved soil moisture dataset by blending passive and active microwave satellite-based retrievals. *Hydrol. Earth Syst. Sci.* **15,** 425–436 (2011).
15. Kirches, G. *et al.* Land cover Climate Change Initiative (CCI). (2014).

16. Tanase, M. A. *et al.* Sensitivity of L-Band Radar Backscatter to Forest Biomass in Semiarid Environments: A Comparative Analysis of Parametric and Nonparametric Models. *Ieee Trans. Geosci. Remote Sens.* **52,** 4671–4685 (2014).

17. Mitchard, E. T. A. *et al.* Using satellite radar backscatter to predict above-ground woody biomass: A consistent relationship across four different African landscapes. *Geophys. Res. Lett.* **36,** (2009).

18. Frost, P. in *The Miombo in transition: woodlands and welfare in Africa* (ed. Campbell, B.) 11–55 (CIFOR, 1996).

19. Willcock, S. *et al.* Quantifying and understanding carbon storage and sequestration within the Eastern Arc Mountains of Tanzania, a tropical biodiversity hotspot. *Carbon Balance Manag.* **9,** 2 (2014).
