# Supplementary material for: Carbon losses from deforestation and widespread degradation offset by extensive growth in African woodlands
Source: Nat Commun. 2018 Aug 2;9:3045. doi: 10.1038/s41467-018-05386-z (PMC6072798; doi:10.1038/s41467-018-05386-z)
Supplement: Supplementary file 3 — Description of Additional Supplementary Files [file 41467_2018_5386_MOESM3_ESM.pdf]

Description of Additional Supplementary Files

File Name: Supplementary Data 1
Description: Supplementary Data 1 contains data on above-ground woody carbon stocks for 2007 - 2010, and the areal extent of each land cover change at the national level, and the two lower administrative units, termed regional (‘province’ in Angola, Mozambique and Zimbabwe; ‘district’ in DRC) and district level (‘municipality’ in Angola; ‘commune’ or ‘territory’ in DRC)."
